# Generation of a monoclonal antibody against duck circovirus capsid protein and its potential application for native viral antigen detection

**DOI:** 10.3389/fmicb.2023.1206038

**Published:** 2023-06-23

**Authors:** Jinxin Li, Fengli Liu, Zhihao Ren, Guanghua Fu, Jizhen Shi, Naiyu Zhao, Yu Huang, Jingliang Su

**Affiliations:** ^1^Key Laboratory of Animal Epidemiology and Zoonosis of the Ministry of Agriculture, College of Veterinary Medicine, China Agricultural University, Beijing, China; ^2^Institute of Animal Husbandry and Veterinary Medicine, Fujian Academy of Agricultural Sciences, Fuzhou, China

**Keywords:** duck, circovirus, DuCV, capsid protein, monoclonal antibody, *in vitro* culturing

## Abstract

**Introduction:**

Duck circovirus (DuCV) infection is currently recognized as an important immunosuppressive disease in commercial duck flocks in China. Specific antibodies against DuCV viral proteins are required to improve diagnostic assays and understand the pathogenesis of DuCV infection.

**Methods and results:**

To generate DuCV-specific monoclonal antibodies (mAbs), a recombinant DuCV capsid protein without the first 36 N-terminal amino acids was produced in *Escherichia coli*. Using the recombinant protein as an immunogen, a mAb was developed that reacted specifically with the DuCV capsid protein, expressed in *E. coli* and baculovirus systems. Using homology modeling and recombinant truncated capsid proteins, the antibody-binding epitope was mapped within the region of ^144^IDKDGQIV^151^, which is exposed to solvent in the virion capsid model structure. To assess the applicability of the mAb to probe the native virus antigen, the murine macrophage cell line RAW267.4 was tested for DuCV replicative permissiveness. Immunofluorescence and Western blot analysis revealed that the mAb recognized the virus in infected cells and the viral antigen in tissue samples collected from clinically infected ducks.

**Discussion:**

This mAb, combined with the *in vitro* culturing method, would have widespread applications in diagnosing and investigating DuCV pathogenesis.

## 1. Introduction

Duck circovirus (DuCV) was first discovered by Hattermann et al. in 2003 from a Mulard duck exhibiting feathering disorder, poor body condition, and low weight (Hattermann et al., 2003). Genomic analysis assigned the isolate to the genus *Circovirus* of the family Circoviridae. Subsequent DuCV infections have been detected in commercial duck flocks in the United States, Europe, and Asia (Banda et al., 2007; Cha et al., 2013; Matczuk et al., 2015; Neale et al., 2022; Tran et al., 2022). DuCV is an important co-factor in duck enteritis virus, goose parvovirus, and *Riemerella anatipestifer* infections (Neale et al., 2022), but the mechanism by which the virus causes the disease is not fully understood. DuCV infection combined with goose parvovirus increases the prevalence of short beak and dwarfism syndrome in meat-type ducks (Liu et al., 2020). The disease induced by DuCV may result from virus-induced immunopathological disorders. In experimentally infected ducks, DuCV targeted the bursa of Fabricius, thymus, and spleen, causing extensive damage to immune organs, and viral DNA was also detected in the liver and intestines (Hong et al., 2018). In the last decade, high rates of DuCV infection in Chinese commercial duck populations have been detected by PCR, with a prevalence of 10–81.6% (Liu et al., 2009; Wang et al., 2011). This high prevalence and the easy circulation of DuCV are a problem for commercial duck flocks. Currently, there is no commercially available vaccine or specific measures to prevent the spread of the disease. Routine diagnostics of DuCV are required to monitor infection numbers in duck flocks and advance our understanding of the pathogenesis of virus infection.

DuCV is a non-enveloped virus with a circular single-stranded genomic DNA that ranges from 1987 to 1996 nucleotides (nt) in length (Breitbart et al., 2017; Liao et al., 2022). The DuCV genome contains at least two major open reading frames (ORFs) encoding the replication-associated protein (Rep) and capsid protein (Cap). Initial structural insights detailing the three-dimensional (3D) structures of circovirus virions used electron microscopy (EM). These EM images revealed that the members of the genus *Circovirus* adopt a similar capsid structure that forms multisubunit virions with T = 1 icosahedral symmetry (Crowther et al., 2003). The first high-resolution atomic structure of PCV2 Cap was solved using X-ray crystallography, showing that the Cap subunits comprise a single canonical jelly roll domain. The jelly roll domain comprises two β-sheets, each containing four β-strands. Shorter loops that connect the β-strands decorate the surface of the capsid, whereas the longer loops predominantly mediate interactions between capsids (Khayat et al., 2011, 2019). Cap is the major structural protein that self-associates to form the capsid of the virus and represents the primary target for antibodies in infected individuals. Thus, Cap is the most frequently used antigen for developing serological diagnostic tests (Liu et al., 2010; Yang et al., 2017).

Monoclonal antibodies (mAbs) against porcine circovirus and psittacine beak and feather disease virus and mAb-based assays have been described (Ritchie et al., 1992; McNeilly et al., 2001). The reported use of mAbs to detect the PCV antigen in cryostat sections from a pig experimentally infected with the virus enabled the identification of the sites of replication of PCV. The mAbs for the psittacine beak and feather disease virus were used to detect PBFDV by immunohistochemistry in related studies (Allan et al., 1994). However, no report has yet described the use of a mAb against DuCV. In this study, a mAb against *Escherichia coli*-expressed DuCV Cap was generated and characterized. Furthermore, *in vitro* culturing of DuCV using the murine macrophage cell line RAW267.4 was investigated. The reactivity of the mAb with native viral Cap in DuCV-infected RAW cells and tissues from clinically diseased ducks was evaluated.

## 2. Materials and methods

### 2.1. Detection of the DuCV genome by PCR

DNA was extracted from duck spleen tissues using a virus DNA kit (Tiangen Co., Ltd, Beijing, China). The PCR detection primers and thermal cycling were performed as described previously (Li et al., 2014). One of the DuCV-positive spleen samples from a Pekin duckling was used to amplify the full-length genome with overlapping primers DuCV-CX-F (5′-TGGCTCTCTCGTGCCCGGGGATCT-3′) and DuCV-CX-R (5′-AGGCTCTTCCTCCCAGCGACT-3′). The sequenced genome of the strain SXD1 was deposited in GenBank.

### 2.2. Generation and purification of recombinant DuCV capsid protein in *E. coli*

There are two reports on the *in vitro* culturing of DuCV (Mészáros et al., 2014; Li et al., 2020), both of which employed methods that require specialized cell lines or techniques. The yield using either of these methods is low. In contrast, the high-level expression of full and truncated fragments of DuCV Cap has been achieved using *E. coli* and baculovirus-based expression systems (Xiang et al., 2013; Lu et al., 2014). In this study, a 684-bp DNA fragment of the Cap encoding gene was amplified with the primers (DuCVorf2-F1: 5′-CGC*GGATCCTTTTCCGTAGTGACATATAA*-3′; DuCVorf2-R1: 5′-CCC*AAGCTT*CTAGAAGCCCGTGAACTGTCC-3′; underlined text shows the restriction endonuclease sequences), using viral DNA as the template. The PCR product was digested with *Bam*HI/*Hin*dIII and cloned into pMAL-C5x and pET-32a vectors. The recombinant plasmids, designated as pMAL-CapΔNLS and pET-CapΔNLS, were confirmed by sequencing and transformed into *E. coli* BL21 cells for protein expression. The recombinant bacteria were propagated in Luria–Bertani (LB) medium containing 100 μg/ml of ampicillin at 37°C with orbital rotation until an OD_600_ of 0.6 was reached. Recombinant protein expression was induced by the addition of 1.0 mM isopropyl-b-D-thiogalactopyranoside (IPTG). Cells were grown for a further 5 h at 37°C. Target protein expression was analyzed by sodium dodecyl sulfate-polyacrylamide gel electrophoresis (SDS-PAGE) and Coomassie brilliant blue gel staining and Western blotting with a monoclonal antibody against the His-tag. His-tagged recombinant proteins were purified by immobilized metal affinity chromatography (Ni-NTA Sepharose) under denaturing conditions and according to the manufacturer's instructions (Solarbio, Beijing, China).

### 2.3. Generation of a eukaryotic expression vector of the DuCV capsid protein

The eukaryotic expression system was constructed by amplifying the 724-bp DNA fragment of the Cap encoding gene using the primers DuCVorf2-F2 (5′-AAAACTGCAGGTGGCGGACCGAAATAAAATGTTTTCCGTAGTGACATATAAGG-3′) and DuCVorf2-R2 (5′-CCGCTCGAGTCAGTG ATGATGATGATGATGGAAGCCCGTGAACTGTCCAAATT-3′), as described above. The amplified fragment was cloned into the *Pst*I/*Xho*I site of the pFastBac baculovirus transfer vector, as described previously (Liu et al., 2021). After confirming the sequence, the pFastBac-CapΔNLS construct was transformed into DH10Bac competent *E. coli* cells (Thermo Fisher Scientific Inc., MA, United States) to produce a recombinant bacmid, according to the manufacturer's instructions. The recombinant bacmid was then purified and transfected into Sf9 cells to produce a recombinant baculovirus containing the DuCV *cap*Δ*NLS* gene.

### 2.4. Generation and screening of DuCV capsid protein-specific hybridoma

Five BALB/c mice (6–8 weeks old; Beijing Vital River Laboratory Animal Technology Co., Ltd., Beijing, China) were immunized subcutaneously with 100 μg of purified *E. coli-*expressing capsid protein pMAL-CapΔNLS with Freund's complete adjuvant. The mice were boosted twice at 2-week intervals with the recombinant capsid protein with Freund's incomplete adjuvant. Two weeks after the third immunization, sera were collected and assessed by indirect ELISA using the following approach. Mice were injected intraperitoneally with 110 μg of the purified recombinant capsid protein. After 3 days, mice were euthanized, and spleen cells were fused with SP2/0 myeloma cells by adding polyethylene glycol 2000. The fused cells were cultured in DMEM containing HAT and 15% fetal bovine serum (FBS), with a layer of macrophage feeder cells collected from a mouse peritoneal cavity. After 10 days of incubation, the hybridoma supernatants were screened by ELISA. ELISA-positive hybridoma cells were then subcloned by limiting dilution. The positive hybridoma clone was expended in a 6-well plate, and the supernatant of the culture was collected for immunoglobulin isotyping using the Mouse Monoclonal Antibody Isotyping Kit (Sigma–Aldrich, St Louis, MO, USA), according to the manufacturer's instructions. The selected hybridoma was inoculated into adult female BALB/c mice to produce an ascitic fluid antibody.

### 2.5. Western blotting

Protein samples were first separated by 12% denaturing SDS-PAGE and visualized by Coomassie brilliant blue staining. The separated proteins were transferred to a polyvinylidene fluoride (PVDF) membrane for Western blotting. Membranes were incubated overnight in blocking solution [1 × phosphate-buffered saline, 0.05% (v/v) Tween-20 (PBST), 5% (w/v) skim milk powder] at 4°C with gentle rocking. After removal of the blocking solution, the mouse monoclonal antibody was diluted in PBST and incubated for 1 h at room temperature. The membrane was then washed three times with PBST, and HRP-conjugated goat anti-mouse IgG was added at a dilution of 1:5000. After incubation at room temperature for 1 h, the membrane was washed, and the signal was developed using the chemiluminescence substrate (ECL reagent; Cwbiotech, Beijing, China).

### 2.6. Indirect enzyme-linked immunosorbent assay (ELISA)

An indirect ELISA was developed to screen the specific antibodies in the immunized mouse sera and hybridoma supernatants. In brief, 96-well ELISA plates were coated with 2 μg/ml purified *E. coli*-expressed DuCV capsid protein pET-CapΔNLS overnight at 4°C. The plate was blocked with blocking solution at 37°C for 2 h and washed three times with PBST. The hybridoma supernatants were added to each well and incubated at 37°C for 1 h. Mouse sera collected before fusion were serially diluted two-fold from the initial dilution of 1:1250. After washing three times with PBST, 100 μl HRP-conjugated goat anti-mouse IgG (1:5000 in PBST) was added and incubated at 37°C for 1 h. After washing, the substrate TMB (3,3′,5,5′-tetramethylbenzidine) was added (100 μl/well), and the reaction was incubated in darkness for 10 min. The reaction was stopped by the addition of 50 μl 2M H_2_SO_4_, and the absorbance was measured at 450 nm.

### 2.7. Indirect immunofluorescence assay (IFA)

mAb binding to recombinant DuCV capsid protein expressed in baculovirus was examined by the seeding of Sf9 cells (ATCC CRL-1711) in a 96-well culture plate in DMEM/F12 (Hyclone) containing 10% FBS and incubating the cells at 28°C. Cells were infected at 80% confluence with the recombinant baculovirus containing the DuCV *cap-*Δ*NLS* gene at a multiplicity of infection (MOI) of 0.1. After 72 h of incubation at 28°C, cells were fixed with pre-chilled acetone/methanol (1:1) for 20 min at room temperature, followed by saponin permeabilization for 10 min. The plate was washed three times with PBST and incubated with a blocking buffer at 37°C for 30 min. Cell wells were washed, and the diluted murine ascitic mAb was added. After incubation at 37°C for 1 h, cell wells were washed, and DyLight 488 AffiniPure Goat Anti-Mouse IgG (H+L; Earthox, CA, USA) was added at a dilution of 1:1000. After incubation at 37°C for 1 h, cells were washed three times and mounted with DAPI. Fluorescence was recorded under a fluorescence microscope (Olympus Corporation, Japan).

### 2.8. *In vitro* culturing of DuCV

The mouse macrophage cell line RAW267.4 (ATCC TIB-71) was cultured in DMEM supplemented with 10% FBS and 1% penicillin-streptomycin at 37°C in the presence of 5% CO_2_ to develop a convenient *in vitro* DuCV culturing method. The original DuCV-positive spleen tissue was homogenized in DMEM, clarified by centrifugation at 8,000 × *g* for 10 min, and filtered through a 0.22-μm membrane filter. The filtered suspension was inoculated to the cell monolayers with ~80% confluence, and DMEM containing 1% FBS was added after 1 h of incubation at 37°C. At 96 h post-infection, the supernatant was transferred to freshly cultured cell monolayers. This step was repeated three times, and the infectivity of DuCV was assessed by real-time PCR with primers qDuCV-F (5′-TTACAAACCACGCGGGAAGT-3′) and qDuCV-R (5′-CTGGGCGGGTTCATACTTGT-5′), which are based on the conservative region of the Rep gene of DuCV SXD1 (GenBank accession No.: OQ759610). qPCR reactions were performed in a CFX Connect™ Real-time PCR Detect System (Bio-Rad, Hercules, CA, USA) reaction solution (20 μl) containing 8.0 μl ddH_2_O, 10 μl 2 × RealStar Fast SYBR qPCR Mix (GenStar, Beijing, China), 1.0 μl template DNA (extracted from RAW cell lysate), and 0.5 μl of each primer, qDuCV-F and qDuCV-R. Thermal cycler conditions were as follows: 95°C for 2 min at stage 1; 40 cycles of 95°C for 15 s, 60°C for 30 s, and 72°C for 30 s. DuCV-infected RAW267.4 cells were also treated for capsid protein detection by IFA and Western blotting using the mAb and following the abovementioned steps.

## 3. Results

### 3.1. Expression of recombinant DuCV capsid protein

**Figure 1:**
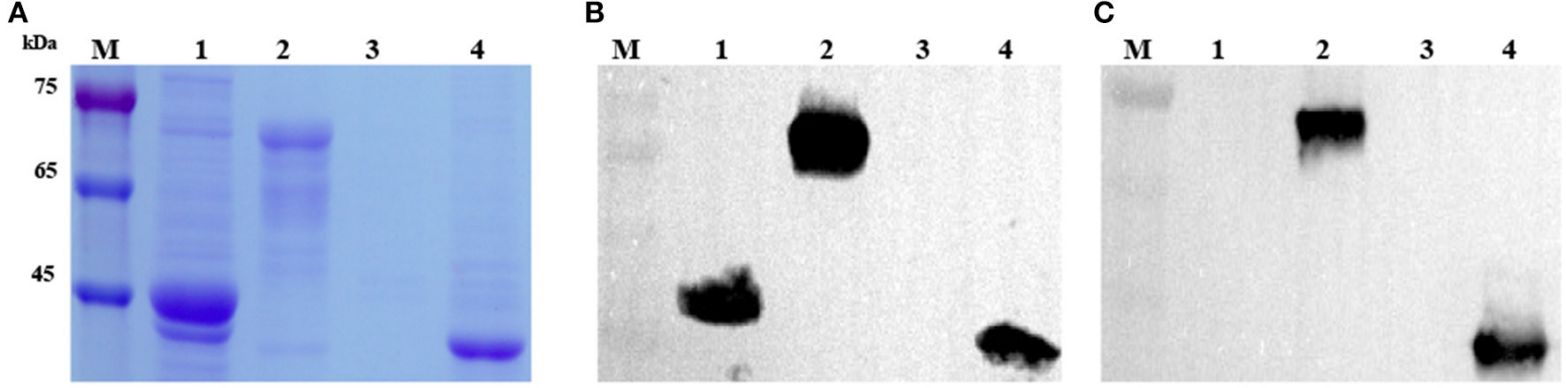
Analysis and identification of recombinant DuCV capsid proteins expressed in *E. coli*. **(A)** SDS-PAGE analysis of recombinant CapΔNLS protein (CP) of DuCV expressed in *E. coli*. **(B)** Western blotting identified the recombinant capsid protein with a monoclonal antibody against the His-tag. **(C)** Western blotting identified the recombinant capsid protein with the monoclonal antibody 4A2. M: protein marker; Lanes 1 and 2: lysates of *E. coli* with the pMAL-C5x vector and pMAL-CapΔNLS; Lanes 3 and 4: lysates of *E. coli* with the pET-32a vector and pET-CapΔNLS.

The target DNA fragment encoding the truncated Cap protein of DuCV was amplified successfully using the DNA template extracted from the DuCV-positive spleen sample. After cloning and transformation, single colonies were selected, and DNA sequence fidelity was confirmed by Sanger sequencing. Constructs transformed into *E. coli* BL21 and protein expression induced by IPTG were assessed by SDS-PAGE. Protein bands in the SDS-PAGE gel corresponding to the expected size (~68.2 kDa for pMAL-CapΔNLS and 40.9 kDa for pET-CapΔNLS) were observed in the cell lysates (Figure 1A). Western blotting showed the recombinant capsid protein reacted with a monoclonal antibody against the His-tag (Figure 1B). His-tagged proteins, pMAL-CapΔNLS and pET-CapΔNLS, were purified using Ni-NTA affinity chromatography and used for mouse immunization and plate-coating antigen in ELISA screening of the monoclonal antibody, respectively.

### 3.2. Generation of a monoclonal antibody against the DuCV capsid protein

MAbs were generated by immunization of mice with recombinant DuCV capsid protein and then tested for the presence of specific serum antibodies. Spleen cells from an immunized mouse were fused with Sp2/0 myeloma cells, and hybridoma supernatants were screened by ELISA using purified pET-CapΔNLS as the plate-coating antigen. After four rounds of sub-cloning, hybridoma 4A2 was eventually established as a monoclonal cell line and chosen for further investigation. The isotype of mAb 4A2 was determined as IgG2a with a kappa light chain. Western blotting showed that the mAb reacted with recombinant DuCV Cap produced in *E. coli* (Figure 1C).

**Figure 2:**
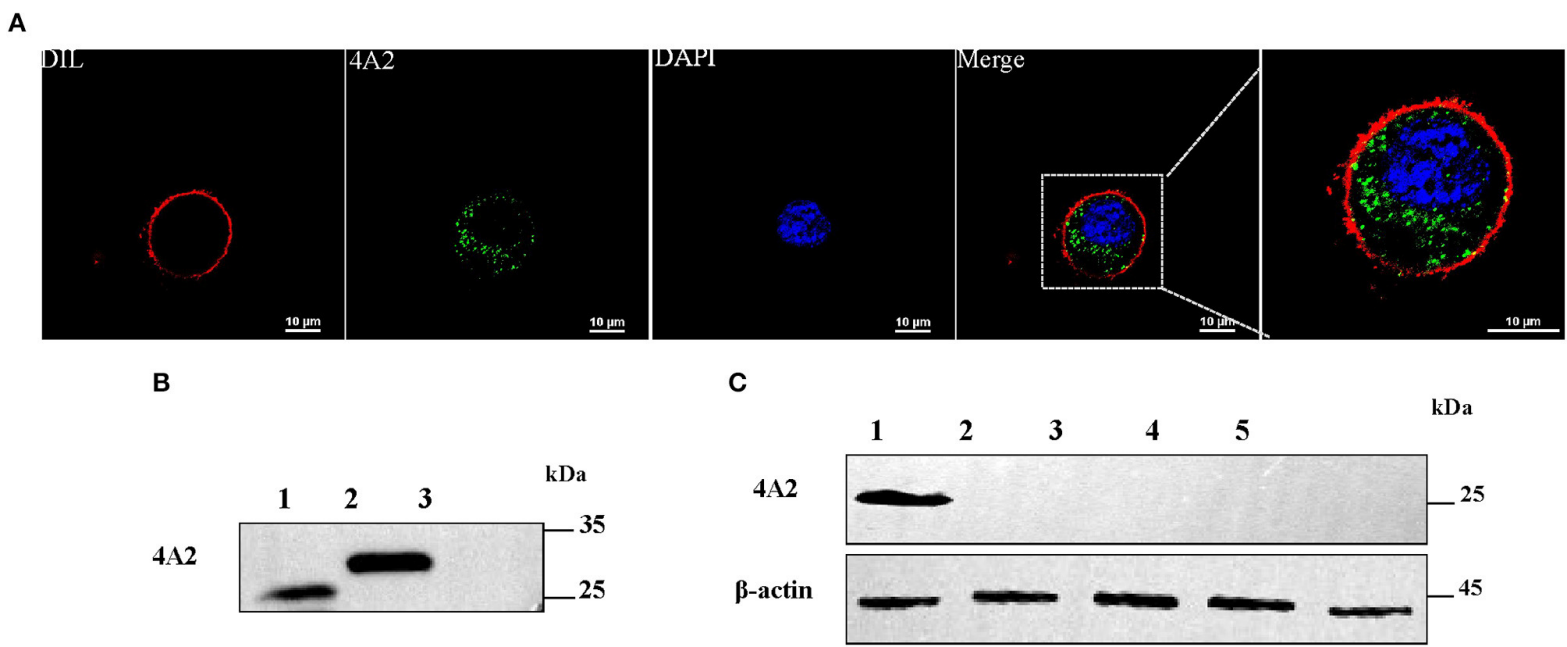
Detection of the reactivity of mAb 4A2 with baculovirus-expressed DuCV capsid protein and different viral proteins by Western blotting. **(A)** IFA staining of Sf9 cells infected with recombinant baculovirus. Cells were infected with recombinant baculovirus containing the DuCV capsid gene. After 72 h post-infection, cells were fixed and labeled with mAb 4A2 and then by the DyLight 488-labeled secondary mAb (green). Nuclei were stained with DAPI (blue), and cytomembranes were stained with DIL (red). **(B)** Western blot analysis of mAb 4A2 with baculovirus-expressed DuCV capsid protein. Lane 1: positive control of recombinant DuCV Cap expressed in duck enteritis virus; Lane 2: protein from the eukaryotic expression of duck circular capsid; Lane 3: protein from Sf9 cells. **(C)** Western blot analysis of mAb 4A2 with different viral proteins. Lane 1: positive control of recombinant DuCV Cap expressed in duck enteritis virus; Lanes 2 to 5: cell lysates of proteins from duck enteritis virus, goose parvovirus, Tembusu virus, and duck astrovirus.

In IFA, mAb 4A2 recognized the DuCV capsid protein, expressed in the recombinant baculovirus-infected Sf9 cells, mainly in the cytoplasm (Figure 2A). Western blot analysis of the infected Sf9 cell lysate using the mAb gave a prominent band with a molecular weight of ~29.1 kDa (Figure 2B). No band was detected in non-infected cells (Figure 2B) or lysates of duck fibroblast cells infected with duck enteritis virus, goose parvovirus, Tembusu virus, or duck astrovirus (Figure 2C). As expected, the cellular marker was detected by an anti-mouse β-actin antibody (Beyotime, Beijing, China). The results indicated that the mAb binds specifically to the DuCV capsid protein.

### 3.3. Recognition epitope of mAb 4A2

**Figure 3:**
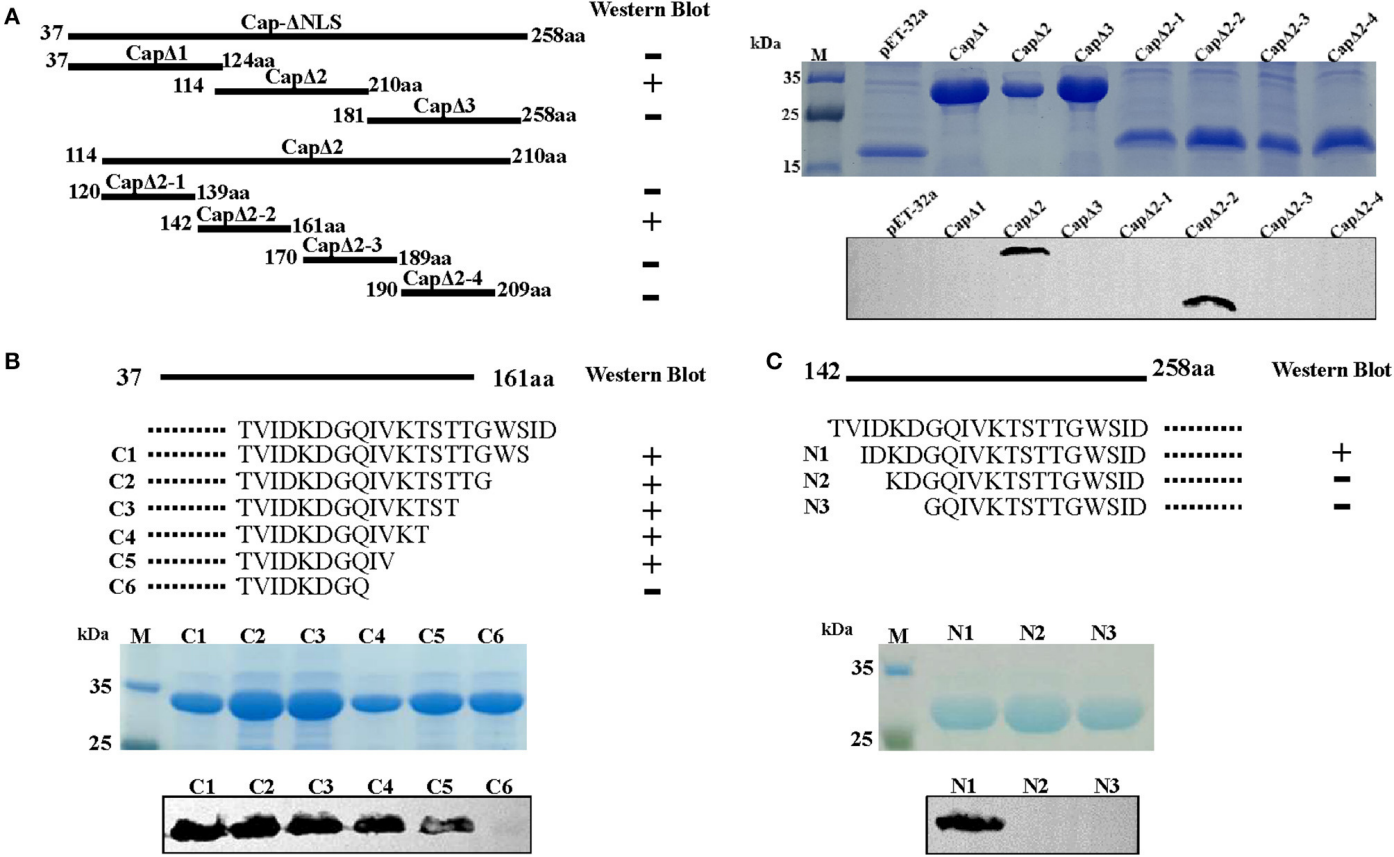
Epitope mapping of mAb 4A2 by Western blot analysis. **(A)** A series of seven overlapping recombinant truncated capsid proteins were synthesized to identify the linear epitope of the mAb. Three N-terminal **(B)** and six C-terminal **(C)** truncated fragments of DuCV capsid were constructed as described in the methods. Western blot analysis indicated that mAb 4A2 specifically reacted with CapΔ2, CapΔ2-2, C1, C2, C3, C4, C5, and N1, of which, the overlapping sequence was ^144^IDKDGQIV^151^.

The epitope region recognized by 4A2 mAb was analyzed by constructing pET-32a-derived recombinant plasmids containing a series of truncated DuCV capsid gene fragments and transformed into *E. coli* BL21. As shown in Figure 3A, truncated Cap proteins were expressed in *E. coli*, and Western blotting analysis showed that CapΔ2 (114–210 aa) and CapΔ2-2 (142–161 aa) reacted with mAb 4A2, presenting a strong signal for the sequence ^142^TVIDKDGQIVKTSTTGWSID^161^. Further omission of amino acid residues from the N- and C-termini of the peptides revealed the sequence ^144^IDKDGQIV^151^ to be essential for 4A2 mAb binding (Figures 3B, C).

Multiple protein sequence alignment of capsid proteins from DuCV, goose circovirus, and pigeon circoviruses indicated that the mapped epitope region ^144^IDKDGQIV^151^ is conserved among DuCV strains, except for strain the TC2 (GenBank: DQ166836), which displays one amino acid substitution, i.e., G148R (Table 1). Amino sequences in the epitope region were noted to be highly divergent between circoviruses from different birds. This divergence was also supported by mAb 4A2 not cross-reacting with the Cap of goose circovirus expressed in *E. coli* (Supplementary Figure 1).

**Table 1:** Multiple protein sequence alignments of the epitope of mAb 4A2 in capsid proteins of DuCV, GoCV, and CoCV (the strains isolated in our laboratory are shown in red).

**Strain name (accession no.)**	**Cap protein**							
	**144**	**145**	**146**	**147**	**148**	**149**	**150**	**151**
DuCV I-SXD1 (OQ759610)	I	D	K	D	G	Q	I	V
DuCV I-JZ22	.	.	.	.	.	.	.	.
DuCV I-YS22	.	.	.	.	.	.	.	.
DuCV I-MH11 (EU344805)	.	.	.	.	.	.	.	.
DuCV I-TC2 (DQ166836)	.	.	.	.	R	.	.	.
DuCV I-LY0701 (EU022374)	.	.	.	.	.	.	.	.
DuCV I-WF0804 (GU131343)	.	.	.	.	.	.	.	.
DuCV II-MH25 (EF451157)	.	.	.	.	.	.	.	.
DuCV II-Germany (AY228555)	.	.	.	.	.	.	.	.
DuCV II-AQ0901 (GU014543)	.	.	.	.	.	.	.	.
DuCV II-WF0801 (GU131340)	.	.	.	.	.	.	.	.
DuCV II-3753-52 (DQ100076)	.	.	.	.	.	.	.	.
DuCV II-ZHEJIANG (GQ334371)	.	.	.	.	.	.	.	.
GoCV-SDJN21-1	I	D	K	E	G	N	I	T
GoCV-DG1 (KT808650)	.	.	.	E	.	N	.	T
GoCV-Hun3 (MG254880)	.	.	.	E	.	N	.	T
GoCV-Anhui15 (MF581299)	.	.	.	E	.	N	.	T
CoCV-Germany (NC002361.1)	P	M	Y	D	A	R	L	K
CoCV-RS0120 (KY114965.1)	P	M	Y	D	A	R	L	K
CoCV-PG-14-844 (KU.593626.1)	P	I	Y	D	A	R	L	K
CoCV-PL14 (MK994769.1)	P	M	Y	D	A	R	L	K
CoCV-TJ62 (MW0253.1)	P	I	Y	D	A	R	L	K

**Figure 4:**
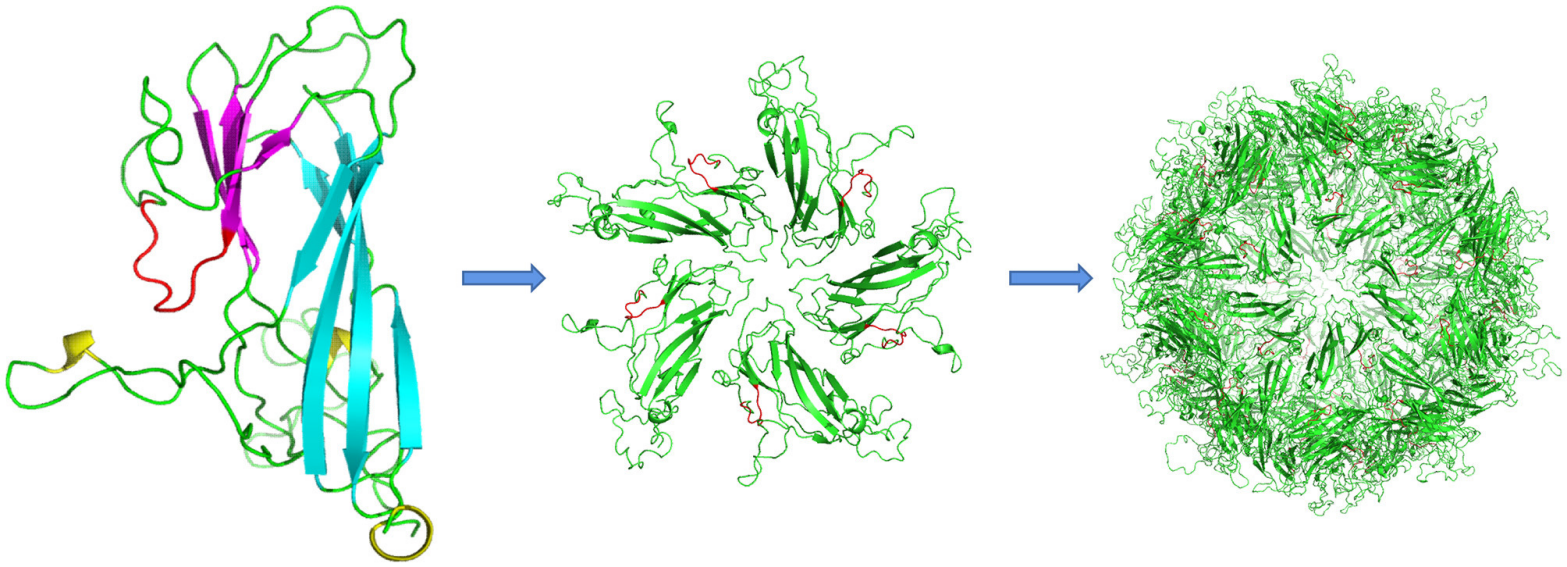
Structural modeling of DuCV Cap. The monomeric unit of the capsid protein comprises two β-sheets composed of five (cyan) and four strands (magenta). The epitope of mAb 4A2 is shown in red, whereas loops are shown in green. One pentamer is shown in the center of each VLP. The epitope region (red) is located immediately after the fifth β-strand and solvent-exposed.

Structural modeling based on the bat circovirus Cap structure (PDB ID: 6rpo.1.A https://swissmodel.expasy.org) indicated that DuCV Cap comprises two β-sheets, one containing five β-strands and the other containing four β-strands, with 12 loops connecting the β-strands. The epitope region was predicted to be positioned immediately after the fifth β-strand and exposed to solvent (Figure 4).

### 3.4. Application of mAb 4A2 for native DuCV antigen detection

**Figure 5:**
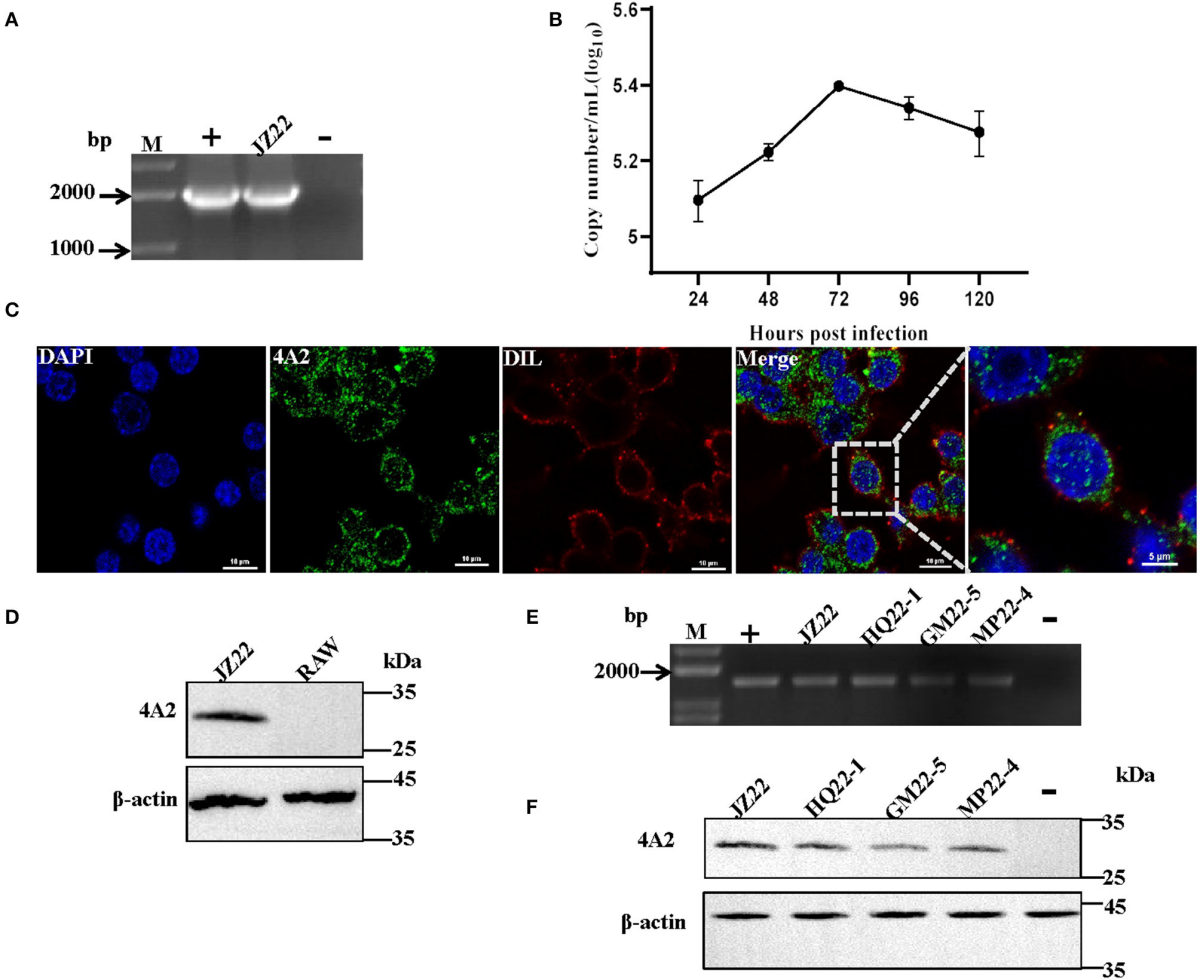
Detection of the native viral antigen of DuCV with mAb 4A2. **(A)** Detection of DuCV strain JZ22 by PCR. **(B)** Growth kinetics of DuCV in RAW cells. **(C)** Immunofluorescence detection of DuCV in RAW cell culture. RAW264.7 inoculated with DuCV strain JZ22 and monolayers were fixed for 72 h post-infection. IFA analysis was performed by probing with mAb 4A2 (green). The nucleus of each cell was stained with DAPI (blue), and the cytomembrane was stained with DIL (red). **(D)** Western blot analysis of a DuCV-infected RAW cell lysate. **(E)** PCR detection of DuCV in clinical duck spleen samples. **(F)** Western blot analysis of the DuCV antigen in clinical duck spleen samples.

To investigate whether the mAb 4A2 can be used for immunological diagnostic methods, IFA was performed on a RAW264.7 cell monolayer infected with DuCV strain JZ22. Replication of DuCV in the cell culture after three sequential passages was detected by PCR (Figure 5A). The kinetic change in the viral DNA copies revealed by real-time PCR indicated modest replication of DuCV in RAW cells (Figure 5B). IFA confirmed the PCR results, with green fluorescence intracytoplasmic foci observed in DuCV JZ22-infected cells (Figure 5C). Further detection of DuCV-infected RAW cell lysates by Western blotting gave a prominent band of ~30 kDa in a dilution of 1:1000 of mAb 4A2 (Figure 5D). No band was detected in the non-infected cell lysate.

Next, we evaluated the diagnostic potential of the generated mAb 4A2. Clinically collected duck spleen samples were detected by Western blotting. Consistent with PCR detection (Figure 5E), the monoclonal antibody detection of the PCR-positive spleen samples at a dilution of 1:100 resulted in a positive band with a molecular weight of ~30 kDa (Figure 5F).

## 4. Discussion

Recent studies have highlighted that the widespread distribution of DuCV infection and its immunosuppressive effects are a problem for commercial duck flocks in China. However, the limited laboratory host system for DuCV propagation hampers the investigation of this virus. More sensitive, specific, and readily standardized diagnostic assays are urgently needed. Applications of mAbs to porcine circoviruses and the beak and feather disease virus have been presented (Ritchie et al., 1992; McNeilly et al., 2001). These applications include using mAbs for immunostaining cell cultures and tissue sections and detecting a virus-specific antibody by ELISA and HI (Shearer et al., 2008). In this study, we reported the generation of a mAb against the DuCV capsid protein in combination with the propagation of a DuCV strain in a RAW cell line. The results should enable further development of standardized immune diagnostic methods.

Only one report has described the *in vitro* culturing of DuCV using continuous cell lines created from primary Muscovy duck retina and somite cells (Mészáros et al., 2014). The relatively low viral productivity and restricted availability of avian cell lines hamper the production of whole DuCV virus particles in large quantities. In this study, a fragment of the DuCV capsid protein that omits the N-terminal NLS region was produced in *E. coli* and baculovirus expression systems. Using the recombinant protein as an immunogen, the mAb 4A2 against DuCV was produced. The antibody-binding epitope was determined to be ^144^IDKDGQIV^151^ of the capsid protein, whose surface is exposed in the capsid protein model and thus accessible for antibody binding (Figure 4). This result was supported by mAb, recognizing the recombinant capsid protein expressed in baculovirus and the native capsid protein in DuCV-infected RAW267.4 cells in IFA and Western blotting analysis. The specific infectivity detected by qRT-PCR and characterized by IFA indicated that DuCV replicated effectively in RAW267.4 cells, providing an option for developing a more convenient *in vitro* culturing approach for DuCV isolates. Given that it detected viral antigens in the spleen tissues from clinically infected ducks, the mAb 4A2 should have widespread applications in both diagnostic detection and research work.

In summary, our study presents the generation of a monoclonal antibody against the DuCV capsid protein and its potential application in viral antigen detection. The successful *in vitro* culturing of DuCV using RAW267.4 cells and the detection of the viral antigen in infected cells are effective alternative approaches to investigate the replication properties of DuCV.

## Data availability statement

The datasets presented in this study can be found in online repositories. The names of the repository/repositories and accession number(s) can be found at: https://www.ncbi.nlm.nih.gov/nuccore/OQ759610.1/.

## Ethics statement

The animal study was reviewed and approved by the China Agricultural University Animal Ethics Committee and it was conducted in accordance with the guidelines of the Beijing Municipality on the Review of Welfare and Ethics of Laboratory Animals, approved by the Beijing Municipality Administration Office of Laboratory Animals and the Regulations for the Administration of Affairs Concerning Experimental Animals, approved by the State Council of China.

## Author contributions

JL and JSu conceived and designed the experiments and wrote the manuscript. JL, FL, ZR, JSh, and NZ performed the experiments. JL, FL, and JSu analyzed the data. GF and YH contributed to reagents/materials. All authors contributed to the article and approved the submitted version.
