# Supplementary material for: Generation of a monoclonal antibody against duck circovirus capsid protein and its potential application for native viral antigen detection
Source: Front Microbiol. 2023 Jun 23;14:1206038. doi: 10.3389/fmicb.2023.1206038 (PMC10326623; doi:10.3389/fmicb.2023.1206038)
Supplement: Supplementary file 2 [file Data_Sheet_1.docx]

Supplementary Material

**Generation of a monoclonal antibody against duck circovirus capsid protein and its potential application for native viral antigen detection**

Jinxin Li^1^, Fenli Liu^1^, Zhihao Ren^1^, Guanghua Fu^2^, Jizhen Shi^1^, Naiyu Zhao^1^, Yu Huang^2*^ and Jingliang Su^1*^

^1^Key Laboratory of Animal Epidemiology and Zoonosis of the Ministry of Agriculture, College of Veterinary Medicine, China Agricultural University, Beijing, China

^2^Institute of Animal Husbandry and Veterinary Medicine, Fujian Academy of Agricultural Sciences, Fuzhou, China

***Correspondence:**Jingliang Su, [suzhang@cau.edu.cn](mailto:suzhang@cau.edu.cn)

Yu Huang, huangyu-815@163.com

# Supplementary Material

***Generation and purification of recombinant GoCV capsid protein in E. coli***

In this study, a 684-bp DNA fragment of the Cap encoding gene was amplified with the primers GoCVorf1-F1 (5′-CGC*GGATCC*CTACACATCGGACGAATAAGG-3′; underlined text represents the restriction endonuclease sequence) and GoCVorf1-R1 (5′-CCC*AAGCTT*TTATGGTGCAAGCCCAGTCCA-3′) using viral DNA (SDJN21) as the template. The PCR product was digested with *Bam*HI/*Hin*dIII and cloned into vector pMAL-C5x. The recombinant plasmid, designated pMAL-Cap*Δ*NLS (GoCV), was confirmed by sequencing and transformed into *E. coli* BL21 cells for protein expression. Recombinant bacteria were propagated in Luria-Bertani (LB) medium containing 100 μg/mL ampicillin at 37℃ with orbital rotation until an OD_600_ of 0.6 was reached. Protein expression was then induced by adding isopropyl-β-D-thiogalactopyranoside (IPTG) to a final concentration of 1 mM. Cells were grown for a further 5 h at 37℃. Target protein expression was analyzed by sodium dodecyl sulfate-polyacrylamide gel electrophoresis (SDS-PAGE) and Coomassie brilliant blue staining. His-tagged recombinant proteins were purified by immobilized metal affinity chromatography (Ni-NTA Sepharose) under denaturing conditions and according to the instructions of the manufacturer (Solarbio, Beijing, China).

## Supplementary Figures


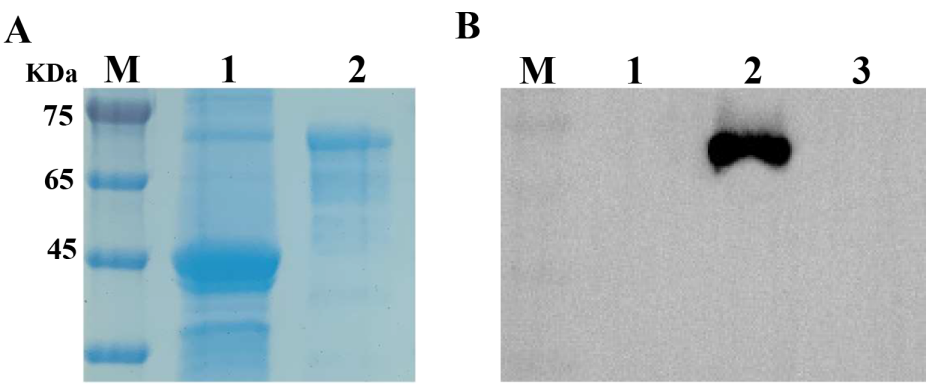
**SUPPLEMENTARY FIGURE 1.** Analysis and identification of recombinant GoCV capsid proteins expressed in *E. coli*. **(A)** SDS-PAGE analysis of the recombinant Cap*Δ*NLS protein (CP) of GoCV expressed in *E. coli*. M: protein marker; Lanes 1 & 2: lysates of *E. coli* with the pMAL-C5x vector and pMAL-Cap*Δ*NLS (GoCV). **(B)** Western blot analysis identified the recombinant capsid protein with monoclonal antibody 4A2. M: protein marker; Lanes 1 to 3: lysates of *E. coli* with the pMAL-C5x vector, pMAL-Cap*Δ*NLS (DuCV) and pMAL-Cap*Δ*NLS (GoCV).
